# Prevalence and genetic diversity of clinical *Vibrio parahaemolyticus* isolates from China, revealed by multilocus sequence typing scheme

**DOI:** 10.3389/fmicb.2015.00291

**Published:** 2015-04-09

**Authors:** Dongsheng Han, Hui Tang, Chuanli Ren, Guangzhou Wang, Lin Zhou, Chongxu Han

**Affiliations:** ^1^Department of Clinical Microbiology, Clinical Medical Examination Center, Northern Jiangsu People's HospitalYangzhou, China; ^2^Department of Biobank, Northern Jiangsu People's HospitalYangzhou, China

**Keywords:** *Vibrio parahaemolyticus*, multilocus sequence typing, phylogenetic analysis, clonal complex, peptide sequence types

## Abstract

The population structure of clinical *Vibrio parahaemolyticus* isolates spreading in China remains undefined. We brought 218 clinical isolates from the pubMLST database originating from different regions of China collected since the year of 1990, analyzed by multilocus sequence typing (MLST), to elucidate the prevalence and genetic diversity of *V. parahaemolyticus* circulating in Chinese population. The MLST scheme produced 137 sequence types (STs). These STs were clustered into six clonal complexes (CCs), six doublets, and 91 singletons, exhibiting a high level of genetic diversity. However, less diversity was displayed on the peptide level: only 46 different peptide sequence type (pST) were generated, with pST2 (44.0%, 96/218) and pST1 (15.1%, 33/218) the predominant. Further analysis confirmed all the pSTs belong to a single complex founded by pST1, pST2, pST3, and pST4. *recA* presented the highest degree of nucleotide diversity (0.026) and the largest number of variable sites (176) on the nucleotide level. *pyrC* was the most diverse locus on the peptide level, possessing the highest percentage of variable sites (9.2%, 15/163). Significant linkage disequilibrium with the alleles was detected when the Standardized Index of Association (*I^S^_A_*) was calculated both for the entire isolates collection (0.7169, *P* < 0.01) and for the 137 STs (*I^S^_A_* = 0.2648, *P* < 0.01). In conclusion, we provide an overview of prevalence and genetic diversity of clinical *V. parahaemolyticus* spreading in Chinese population using MLST analysis. The results would offer genetic evidences for uncovering the microevolution relationship of *V. parahaemolyticus* populations.

## Introduction

*Vibrio parahaemolyticus* is a leading cause of food-borne outbreaks and acute gastroenteritis throughout the world, especially in coastal countries and regions. Consumption of raw or undercooked seafood is the major route of transmission for *V. parahaemolyticus* infection (Pal and Das, 2010). *V. parahaemolyticus* infection is caused by diverse serotypes; however, since the pandemic serovar O3:K6 emerged in Asia in 1996, it was confirmed as the predominant cause of outbreaks of *V. parahaemolyticus* infection on a global scale (Okuda et al., 1997; Bag et al., 1999; Chowdhury et al., 2000; Nair et al., 2007). In recent years, at least 21 pandemic serotypes were identified as being associated with the outbreaks of *V. parahaemolyticus* infection (Nair et al., 2007).

*V. parahaemolyticus* is the first leading cause of food-borne outbreaks and bacterial infectious diarrhea in China, especially in the southeast coastal area (Lin et al., 2011). During 2000–2009, a multicentric surveillance for *V. parahaemolyticus* diarrhea at 11 provinces (Beijing, Jiangsu, Shanghai, Zhejiang, et al.) was conducted by China CDC, they collected stool or rectal swab specimens from 79,075 diarrhea patients to detected the prevalence ratio of *V. parahaemolyticus*, and found the average ratio of *V. parahaemolyticus* was 3.11% in these diarrhea patients. Studies also confirmed clinical isolates in China typically correspond to the pandemic serovar O3:K6 and several serovariants of it (e.g., O1:KUT, O1:K56, and O4:K68) (Chao et al., 2009; Yu et al., 2011; Fan et al., 2013; Shi et al., 2013; Li et al., 2014). So it's critical to clarify the prevalence and genetic diversity of this pathogen circulating in a particular population for minimizing both the risk of infection and economical burden. For the molecular genetic studies of *V. parahaemolyticus*, a number of molecular typing techniques have been developed and applied (Marshall et al., 1999; Gonzalez-Escalona et al., 2008; Chen et al., 2012). Multilocus sequence typing (MLST) of *V. parahaemolyticus* was developed by González-Escalona et al in 2008 (Gonzalez-Escalona et al., 2008). Gonzalez-Escalona's own and a number of subsequent studies demonstrated MLST was a powerful tool with a high resolution rate in identification of clonal complexes (CCs) of *V. parahaemolyticus* population and in understanding the processes leading to the emergence and spread of pathogenic isolates (Harth et al., 2009; Yu et al., 2011; Ellis et al., 2012; Banerjee et al., 2014).

Although several studies already used MLST analysis to study the genetic diversity of *V. parahaemolyticus* in China in recent years, they were restricted to specific regions (Chao et al., 2009; Han et al., 2012; Fan et al., 2013; Shi et al., 2013), focused exclusively on pandemic pathogenic isolates (Chao et al., 2011; Yan et al., 2011) or were based on a limited isolate number (Yu et al., 2011). In the present study, we brought 218 Chinese clinical isolates from the pubMLST database (http://pubmlst.org/vparahaemolyticus/) into our analyses. These clinical isolates were collected mostly from the provinces of Beijing, Jiangsu, Zhejiang, Fujian, Guangdong, and Taiwan. With these isolates, we aimed to elucidate the prevalence and genetic diversity of clinical *V. parahaemolyticus* isolates circulating in Chinese population. We would investigate the sequence/peptide polymorphisms of the isolates and analyze the probable evolutionary relationships among the isolates. The differences in regard to CC and sequence type (ST)/peptide sequence type (pST) affiliation in the analyzed isolates were considered. We provide a broader overview of the genetic population structure of clinical *V. parahaemolyticus* from China, and predict that the results will provide genetic evidences for uncovering the microevolution relationship among different isolates and might be conducive to the early warning and prevention of *V. parahaemolyticus* infection.

## Materials and methods

### Sampling of *V. parahaemolyticus* isolates

A total of 218 clinical *V. parahaemolyticus* isolates from Chinese patients with acute gastroenteritis were selected as the research subject of this study, they were all available in the pubMLST database (http://pubmlst.org/vparahaemolyticus/). These isolates were both temporally (collected from 1990 to November 2014) and geographically (collected from the provinces of Beijing, Jiangsu, Zhejiang, Fujian, Guangdong, and Taiwan) diverse (see Table S1 in the Supplemental Material).

### MLST analysis and phylogenetic analysis

There is a PCR protocol of internal fragments of the seven housekeeping genes [*recA*_(729*bp*)_, *dnaE*_(557*bp*)_, *gyrB*_(592*bp*)_, *dtdS*_(458*bp*)_, *pntA*_(430*bp*)_, *pyrC*_(493*bp*)_, and *tnaA*_(423*bp*)_] on *V. parahaemolyticus* pubMLST website (http://pubmlst.org/vparahaemolyticus/), the allele designations and STs of all the 218 isolates had been determined. Based on the related STs, all the isolates were subdivided into CCs or groups by goeBURST analysis using Phyloviz software (http://www.phyloviz.net). We also implemented “population snapshot” analysis on the basis of STs and pSTs by using goeBURST. Isolates that shared 100% identity in six of the seven loci with at least one other member of the group, the single locus variants (SLVs), were assigned to a single CC. The primary founder of a CC, SLVs, double locus variants (DLVs), and singletons were defined as in previous study (Han et al., 2014).

When a nucleotide sequence was translated in frame, a peptide sequence could be obtained, in other words, each nucleotide sequence correspond to a unique peptide sequence, an individual isolate contains a unique ST as well as a pST. So translating the in-frame nucleotide sequences into peptide sequences allows a phylogenetic analysis based on pSTs. In this study, the assignment of pSTs of the analyzed isolates to CCs was carried out as previously (Theethakaew et al., 2013; Urmersbach et al., 2014) and predicted also by goeBURST algorithm.

Minimum-evolution (ME) trees for the in-frame concatenated sequences (*recA*-*dnaE*- *gyrB*-*dtdS*-*pntA*-*pyrC*-*tnaA*) of each (p)ST were constructed by Mega 5 software, genetic distance of the analyzed isolates was estimated by the Kimura two-parameter model, as did in the other study (Han et al., 2014).

### Population genetic analysis

DnaSP V5 was used to calculate the following parameters: the number of alleles, the number of polymorphic sites and nucleotide diversity(π), for evaluating the varying degrees of the loci in our selected isolates (Librado and Rozas, 2009). START V2 was implemented to calculate the ratio of non-synonymous-to-synonymous substitutions (d*N*/d*S*) through the Nei and Gojobori method (Jolley et al., 2001). d*N*/d*S* < 1 indicates that the relative gene was mainly affected by purifying selection during the population evolution, d*N*/d*S* = 1 indicates neutral selection and d*N*/d*S* > 1 indicates positive selection. The value of Standardized Index of Association (*I^S^_A_*) was calculated by START2, in order to access the population structure of *V. parahaemolyticus*. *I^S^_A_* = 0 indicates alleles are in linkage equilibrium (alleles are independently distributed at all loci analyzed) and recombination occurred frequently (Gonzalez-Escalona et al., 2008).

## Results

### Diversity of sequence types (STs)

The data on diversities of the seven loci in the 218 *V. parahaemolyticus* isolates are showed in Table 1. All these analyzed isolates resulted in 137 unique STs by applying MLST analysis. Among these STs, Individual STs were mostly recovered once (118 STs), ST3 was most frequent (52 isolates), 18 STs was constituted of between two and five isolates (see Table S1 in the Supplemental Material). When the geographical subsets were considered, the number of different STs was high in Guangdong, Jiangsu, Taiwan and Shanghai (Table 2).

**Table 1:** **Diversities of the seven loci in the 218 *V. parahaemolyticus* isolates**.

**Locus**	**Fragment size**		**No of alleles**		**No (%) of variable sites**		**nucleotide diversity (π)**	**d*N*/d*S***
	**ST**	**pST**	**ST**	**pST**	**ST**	**pST**	**ST**	**ST**
**I**								
*dnaE*	557bp	185aa	67	6	63 (11.31)	5 (2.70)	0.012	0.028
*gyrB*	592bp	197aa	79	2	62 (10.47)	–(–)^*^	0.015	0.001
*recA*	729bp	242aa	75	4	176 (24.14)	19 (7.85)	0.026	0.017
**II**								
*dtdS*	458bp	152aa	68	3	47 (10.26)	2 (1.32)	0.023	0.002
*pntA*	430bp	143aa	51	12	40 (9.30)	10 (6.99)	0.011	0.031
*pyrC*	493bp	163aa	62	16	52 (10.55)	15 (9.20)	0.013	0.066
*tnaA*	423bp	141aa	51	7	42 (9.93)	6 (4.26)	0.013	0.013

* *For the occurring alleles no variable sites could be determined*.

**Table 2:** **Geographic distribution of the 218 *V. parahaemolyticus* isolates**.

**Geographical subsets**	**No. of isolates**	**No. of STs**	**Mainly STs**	**No. of pSTs**	**Mainly pSTs**
Guangdong	51	33	ST3, ST305, ST480, etc.	16	pST1,pST2,pST11, pST116, etc.
Jiangsu	37	22	ST3,ST8,ST120, etc.	14	pST1,pST2,pST4, pST26, etc.
Taiwan	30	11	ST3,ST8, ST120, etc.	7	pST1,pST2,pST4, pST31, etc.
Shanghai	17	17	ST283,ST301,ST302, etc.	6	pST1,pST2,pST3, pST4, etc.
zhejiang	5	5	ST654,ST670,ST671, etc.	3	pST1,pST2,pST16
Beijing	4	4	ST431,ST432,ST478,ST479	3	pST1,pST2,pST163
Hong Kong	3	2	ST3,ST91	2	pST1,pST2
Fujian	2	2	ST963,ST1034	2	pST2, pST197
Unkown	69	53	ST3,ST69,ST216, etc.	21	pST1,pST2,pST3, pST4, etc.
ALL isolates	218	137	ST3,ST8, ST120, etc.	46	pST1,pST2,pST3, pST4, etc.

The number of alleles observed for each locus ranged from 51 (*pntA* and *tnaA*) to 79 (*gyrB*). *gyrB* possessed the most number of alleles (79 alleles) but only 62 (10.47%) variable sites. The nucleotide diversity ranged from 0.011(*pntA*) to 0.026(*recA*). The d*N*/d*S* ratio for every locus was close to zero (Table 1), this suggests that the housekeeping genes are mainly affected by purifying selection during the evolutionary process.

### Diversity of peptide sequence types (pSTs)

A total of 46 different pSTs were obtained from the analyzed isolates (see Table S1 in the supplemental material), occurred with a frequency of 0.5 to 44.0%. pST2 (44.0%, 96/218) and pST1 (15.1%, 33/218) were the two predominant pSTs. One particular pST could be comprised of numerous STs, in this study, we found that pST1 were translated by the nucleotide sequences of 27 different STs (ST62, ST91, ST120, etc.), pST2 by 38 STs (ST3, ST189, ST345, etc.), pST3 by 7 STs (ST328, ST332, ST444, etc.), pST4 by 9 STs (ST8, ST224, ST301, etc.), and the other pSTs by four or less STs.

On pST level, the proportion of allele ONE was more than 90.0% for most of the seven loci, except for *dnaE* (allele ONE accounting for 35.8%) and *pyrC* (allele ONE accounting for 73.4%) (see Table S1 in the Supplemental Material). The individual loci possessed 2 (*gyrB*) to 16 (*pyrC*) unique alleles. *pyrC* possessed the highest percentage of variable sites (9.2%, 15/163) (Table 1).

### Clonal relationships of the collected isolates

In this study, the calculated *I^S^_A_* value was 0.7169 (*P* < 0.01) for all of the 218 analyzed isolates, that is to say, the alleles in the seven housekeeping genes are in linkage disequilibrium. When we calculated the *I^S^_A_* value repeatedly, using one isolate to represent each of the 137 STs, it's 0.2648 (*P* < 0.01). Although the value represented a markedly decrease from 0.7169, these alleles are in linkage disequilibrium. This indicates a nonrandom distribution of alleles in the *V.parahaemolyticus* population in general.

### Identification of clonal complexes

**Figure 1:**
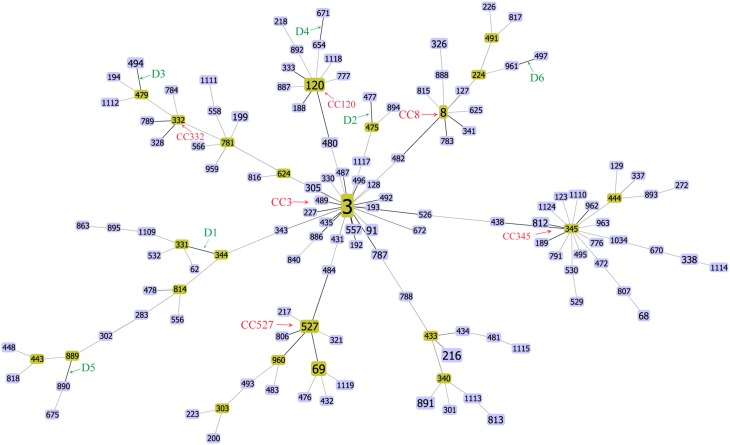
**FullMST of *V. parahaemolyticus* in China based on nucleotide sequence**. Six clonal complexes (CC3, CC8, C120, CC332, CC345, and CC527), six doublets (D1–D6), and 91 singletons were identified. All connections were drawn. STs that are SLVs of each other are connected by black lines. STs that differ in two or more alleles are connected via light gray lines.

**Figure 2:**
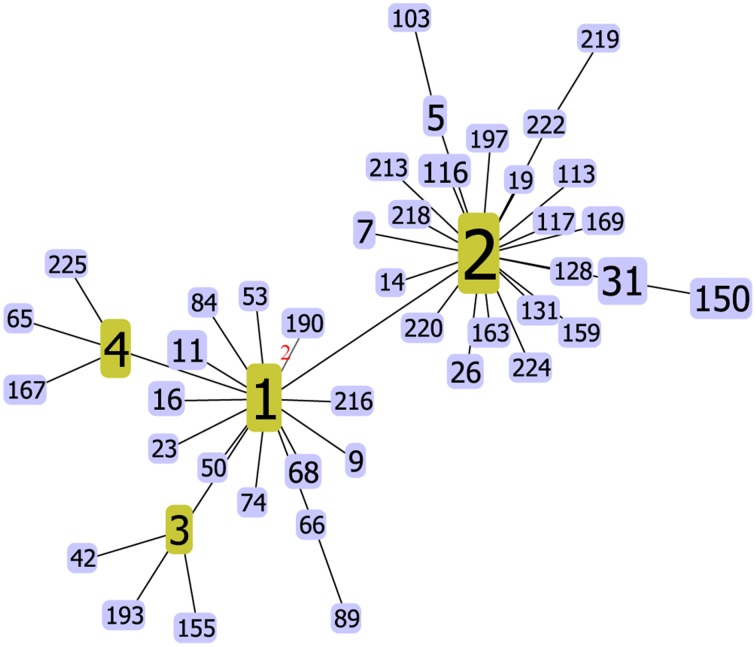
***V. parahaemolyticus* “population snapshot” in China based on peptide sequence**. The number of different alleles is indicated in the case of DLVs, all other pSTs are SLVs. In fact, only pST190 is a DLV of pST1, all other pSTs are SLVs of pST1, pST2, pST3, or pST4.

The goeBURST algorithm used in our study resolved the 137 STs into six CCs (CC3, CC8, C120, CC332, CC345, and CC527) and six doublets (D1–D6). The remaining 91 STs were singletons (Figure 1). CC3 was the most prevalent CC, including 66 isolates with 13 STs. However, neither the relationships between the 91 singletons themselves nor the relationships between the singletons and the defined CCs or doublets could be deduced here. It suggested that goeBURST had limited utility on nucleotide level for identifying related isolates. To counter this, we implemented a “population snapshot” analysis by using goeBURST on the basis of pSTs. The result showed that only pST190 differed in more than one allele to all the other 45 pSTs, leading to a single complex founded by pST1, pST2, pST3, and pST4 (Figure 2). So the relationship among the isolates appears more closely when analyzed on peptide level than on nucleotide level.

### Phylogenetic analysis

**Figure 3:**
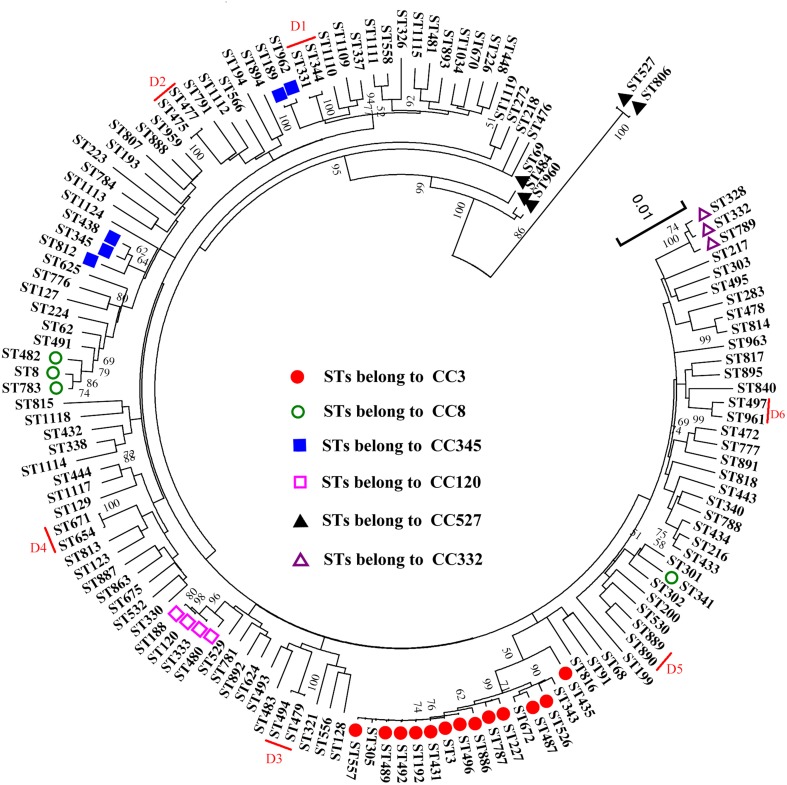
**An ME tree was constructed using the concatenated sequences of the seven loci (*recA*-*dnaE*-*gyrB*-*dtdS*-*pntA*-*pyrC*-*tnaA*) of the 137 STs**. Circles, squares, and triangles with different shading represent the six CCs observed by goeBURST, the six doublets marked by red lines. The scale represents the evolutionary distance. Bootstrap values over 50% are shown on the branches.

The result of a phylogenetic analysis using ME tree on nucleotide level revealed a high genetic diversity among the 218 analyzed isolates (Figure 3). The isolates belong to the same CCs and Doublets (D1–D6) in the goeBURST analysis were also clustered together in the ME tree, except for the isolates of CC345 and CC527. CC345 was divided into two different clusters, ST438 and ST812 exhibited a relatively closer evolutionary distance to ST345 (the ancestral type of CC345) than ST962 and ST189. When compared the number of single nucleotide polymorphisms (SNPs), we found ST962 and ST189 showed differences of 25 SNPs (found in *gyrB* allele), and 24 SNPs (found in *recA* allele) from ST345 respectively, whereas ST438 and ST812 only differs from ST345 by 9 SNPs (found in *recA* allele) and 15 SNPs (found in *recA* allele), respectively. Similarly, the difference between SNP numbers had an important impact on the classification of CC527, ST806 (only one SNP different from ST527) showed a closer evolutionary relationships to ST527 than ST69 (139 SNPs differs), ST484 (136 SNPs differs), and ST960 (135 SNPs differs). Thus, a phylogenetic analysis based on the nucleotide sequences provides a better resolution and elucidated some genetic relationships among isolates that were not resolved by goeBURST analysis.

**Figure 4:**
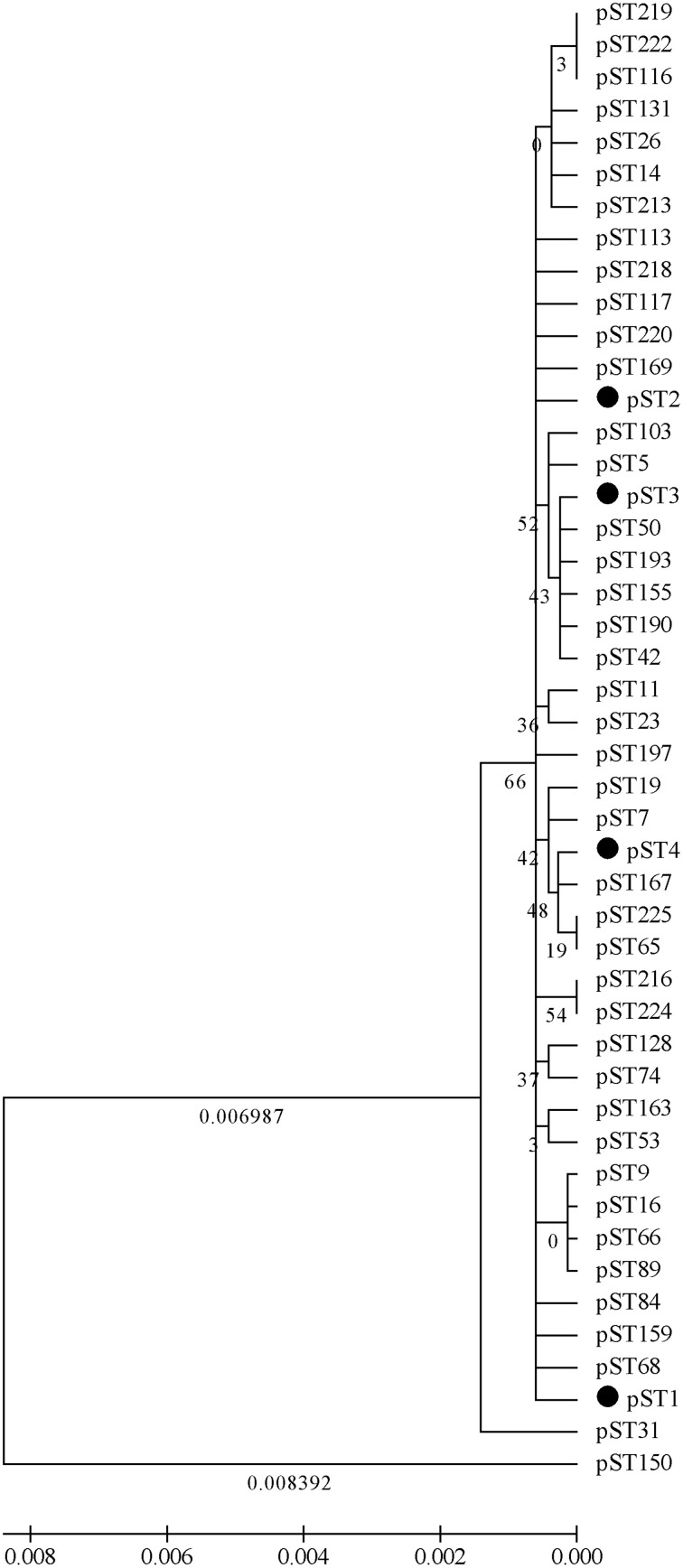
**A ME tree was constructed using the concatenated peptide sequences of the 46 pSTs**. Bootstrap values over 50% and the branch lengths (evolutionary distances) over 0.005 are shown in the figure.

An ME tree analysis was also conducted based on the concatenated sequences of peptide sequences among the 46 pSTs (Figure 4). The low bootstrap values (all<70%) indicated that the topology of the ME tree was poorly supported. However, the longest branch length (evolutionary distance) was only 0.008392 (found between ST150 and the cluster of other pSTs); the genetic similarity of all the clusters of the pSTs was higher than 99.99%. Thus, the 46 analyzed pSTs belong to a single CC when a phylogenetic analysis carried out on peptide level.

## Discussion

In the present study, we analyzed the prevalence and extent of genetic diversity of *V. parahaemolyticus* among 218 clinical isolates collected in different regions of China. The diversity of the *V. parahaemolyticus* isolates was analyzed on nucleotide as well as peptide level. It is clear that multiple sequence/peptide types are contributed to human infection in China, and the genetic relationship among the isolates appears more closely on peptide level than on nucleotide level.

The observed alleles, variable sites, d*N*/d*S*-value, nucleotide diversity (π), *I^S^_A_*-value and (p)STs of our isolates were similar to those derived from a collection of global isolates in the pubMLST database, which were presented in a study conducted by Urmersbach et al. (2014). This reveals a high diversity of clinical isolates collection in Chinese population.

The low d*N*/d*S* ratios (all close to zero) obtained for all the seven genes indicating purifying selection for all the loci, as shown by others (Turner et al., 2013). It means synonymous substitutions were dominant in the nucleotide sequences, which do not alter amino acid sequences. This finding could explain the fact that the numbers of different alleles per locus were reduced and the loci were dominated by a single allele when analyzed on peptide level.

*V. parahaemolyticus* population was extremely genetic diverse in China. A total of 137 STs were identified in this study. ST3 was the most common ST, in agreement with the finding of a previous study on the basis of a global clinical isolates collection (Han et al., 2014). As we know, ST3 has been a sequence type of *V. parahaemolyticus* with an international distribution. Numerous reports revealed that it was widely distributed and played an important role in *V. parahaemolyticus* infection in China (Yu et al., 2011; Han et al., 2012; Fan et al., 2013; Shi et al., 2013). However, when carried out an analysis based on the peptide sequences, the diversity decreased. Only 46 pSTs were generated, and pST1, pST2, pST3, and pST4 were the most prevalent pSTs, which also the predominant pSTs in the pubMLST database (Urmersbach et al., 2014).

Eight CCs and 11 doublets have been identified when we analyzed the clinical isolates with a global collection in the pubMLST dataset (Han et al., 2014). In this study, six CCs and six doublets were found in the “population snapshot” of the 137 STs. CC3, a global pandemic clone of *V. parahaemolyticus* (Martinez-Urtaza et al., 2004, 2005; Ansaruzzaman et al., 2005), was also the most prevalent CC in China, being comprised of 69 isolates with 13 different STs in this study, posing a significant public health threat. However, the “population snapshot” of pSTs consists of only one unique CC. with pST1, pST2, pST3, and pST4 being the ancestral types at the same time. Other pSTs might have arisen from the four types by genetic drift associated with genetic changes (Osorio et al., 2012).

As discussed above, when we analyzed the genetic relationships among different isolates based on STs, goeBURST algorithm showed a decreased ability in identifying the related genotypic relationships due to the high degree of allelic diversity. Relationships are reliable only for identical or closely related isolates. When isolates are more distantly related, such as the 91 singletons, little information can be gained about their relationships. However, when the goeBURST analysis was implemented on the basis of pSTs, all the isolates were classified into one unique CC. This result maybe more representative of the real relationships among the isolates on the phenotypic level. Using pSTs instead of STs might be more efficient in reaching a reliable identification of related isolates.

Previous study confirmed that linkage disequilibrium could be observed when a recent, more epidemic clone arises (Smith et al., 1993). The calculated *I^S^_A_* value was 0.7169 (*P* < 0.01) for all of the analyzed isolates in this study, suggesting that all alleles in the seven housekeeping genes were in linkage disequilibrium or were non-randomly distributed. However, even the analysis was repeated using one isolate to represent each of the 137 STs, which would weaken the influence of the potential pandemic isolates in the data set, these alleles are still in linkage disequilibrium (*I^S^_A_* = 0.2648, *P* < 0.01). These results indicate that a non-randomly distribution of alleles in the *V. parahaemolyticus* population in general, even though recombination might be occurring in different subtypes (Gonzalez-Escalona et al., 2008). These observations are also typical for epidemic populations (Ellis et al., 2012; Theethakaew et al., 2013; Turner et al., 2013), thus to some extent, our data support the hypothesis that the population structure of *V. parahaemolyticus* follows the epidemic model of clonal expansion (Yu et al., 2011).

The results of goeBURST and ME tree shared a high similarity in the identifying of CCs. In the MLST scheme, the isolates of four CCs (CC3, CC8, C120, and CC332) and the six doublets were also clustered together in the ME tree, indicating that they were genetically exclusive complexes or groups. However, the isolates of CC345 and CC527 were resolved into different clusters. It could be explained by the different approaches used in ME tree and goeBRUST algorithm. The ME tree is sequence-based, all sequences with fewer differences could be clustered together. goeBRUST algorithm is allelic profile-based, only the SLVs were assigned to a single CC or groups. So the ME tree seems to be more suitable for analyzing genetic relationships of *V. parahaemolyticus* populations (Yan et al., 2011).

An ME tree was also constructed for phylogenetic analysis of *V. parahaemolyticus* on peptide level. All the 46 analyzed pSTs are grouped into a single CC, with the genetic similarity of all the clusters of the pSTs higher than 99.99%. A similar result has been observed by Osorio et al. who aimed to deduce putative ancestral relationships between different *Brachyspira hyodysenteriae* isolates (Osorio et al., 2012).

In this study, isolates without a clear source or STs were screened out. This may has some influence on the genetic characteristics of *V. parahaemolyticus* in China in general. However, our findings would facilitate the researchers in this field to understand the population structure of *V. parahaemolyticus* in China. As the *V. parahaemolyticus* PubMLST database is not mandatory for uploading laboratory data, not all the research data of *V. parahaemolyticus* in the world (including China) have been completely uploaded into this public database. Only when a new ST is discovered, the researcher will sent the isolate information to the manager of the database to get identification for a new subtype. Therefore, the pubMLST database contains all the STs of *V. parahaemolyticus* all around the world, but does not contain all the discovered isolates. Here, we recommend that the database should be made some mandatory improvement, for example, when a new subtype is uploaded, the corresponding biological characteristics of isolates (sample source, resistance, serotype, virulence gene, etc.) should be also updated. We believe this will enable the pubMLST database to play a more important role in studies on infection and molecular epidemiology of *V. parahaemolyticus*.

In summary, we provide an overview of prevalence and genetic diversity of clinical *V. parahaemolyticus* spreading in Chinese population using MLST analysis. We implemented the identification of CCs and phylogenetic analysis both on nucleotide level and on peptide level. The results in this study will provide genetic evidences for uncovering the microevolution relationship among different pathogenic isolates of *V. parahaemolyticus*. The pubMLST database provides a platform for the comprehensive analysis of genetic relationships of *V. parahaemolyticus*. With the growing number of the uploaded isolates, more molecular biological characteristics of *V. parahaemolyticus* in china and other counties would be revealed.

### Conflict of interest statement

The authors declare that the research was conducted in the absence of any commercial or financial relationships that could be construed as a potential conflict of interest.
